# Small noncoding RNA profiles along alternative developmental trajectories in an annual killifish

**DOI:** 10.1038/s41598-018-31466-7

**Published:** 2018-09-06

**Authors:** Amie L. T. Romney, Jason E. Podrabsky

**Affiliations:** 1 Department of Biology, Portland State University, P.O. Box 751, Portland, OR 97207 USA; 2 Department of Anatomy, Physiology & Cell Biology, University of California at Davis School of Veterinary Medicine, One Shields Ave, Davis, CA 95616 USA

## Abstract

Embryonic development of *Austrofundulus limnaeus* can occur along two phenotypic trajectories that are physiologically and biochemically distinct. Phenotype appears to be influenced by maternal provisioning based on the observation that young females produce predominately non-diapausing embryos and older females produce mostly diapausing embryos. Embryonic incubation temperature can override this pattern and alter trajectory. We hypothesized that temperature-induced phenotypic plasticity may be regulated by post-transcriptional modification via noncoding RNAs. As a first step to exploring this possibility, RNA-seq was used to generate transcriptomic profiles of small noncoding RNAs in embryos developing along the two alternative trajectories. We find distinct profiles of mature sequences belonging to the miR-10 family expressed in increasing abundance during development and mature sequences of miR-430 that follow the opposite pattern. Furthermore, miR-430 sequences are enriched in escape trajectory embryos. MiR-430 family members are known to target maternally provisioned mRNAs in zebrafish and may operate similarly in *A. limnaeus* in the context of normal development, and also by targeting trajectory-specific mRNAs. This expression pattern and function for miR-430 presents a potentially novel model for maternal-embryonic conflict in gene regulation that provides the embryo the ability to override maternal programming in the face of altered environmental conditions.

## Introduction

The genetic toolkit for vertebrate development provides the evolutionary foundation that supports diverse phenotypic outcomes^1,2^. However, the contents of this toolkit remain poorly understood. Patterns of gene transcription during early development are highly conserved, but alterations in the timing and especially the level of expression of genes (i.e. control of gene *activity*) during early developmental stages are thought to underlie species-specific morphogenetic processes, while still allowing for “normal” development of key features and structures^3^. Conserved gene expression programs can be altered post-transcriptionally through a variety of mechanisms including gene regulatory networks of small RNAs. Regulation of gene expression by small non-coding RNAs (ncRNAs) has emerged as an important driver in the generation of phenotypic complexity^4,5^. Here we explore the potential for small ncRNAs to contribute to the developmental program and phenotypic plasticity during development of the annual killifish *A. limnaeus*.

Modification of mRNA structure and stability are especially important steps at which gene expression can be regulated, and can lead to significant variations in sequence and structure of mRNAs from a single genomic region (gene). Understanding the mechanisms of how RNAs are processed, what factors determine their stability, translatability, and thus their ultimate expression could explain such phenomena as the expression of alternative developmental phenotypes generated from a single genetic background. Small ncRNAs can regulate chromatin structure as well as induce RNA degradation and translational repression by serving as RNA scaffolds that can target specific nucleotide sequences for alteration by a variety of partner protein complexes^6^. This unifying mechanism for the action of small ncRNAs, and an apparent high degree of evolutionary conservation of function, allows for the identification of potential gene targets of small ncRNAs during vertebrate development.

A small number of small ncRNA classes have widely recognized roles in directing gene regulation during animal development and include microRNA (miRNA), antisense RNA (asRNA), and piwiRNA (piRNA)^7–11^. Among the various classes, miRNAs are perhaps best known for their role controlling developmental timing of the nematode, *Caenorhabditis elegans*^12^. After being processed from double-stranded precursor RNAs ~80 nucleotides (nts) in length, miRNAs typically have a mature length of approximately 22 nts. They bind to an argonaute protein to form the RNA-induced silencing complex (RISC)^13–16^. Acting as sequence specific guides, complementary base pairing occurs between the miRNA “seed” (nts 2–7 on the 5′ end) and sites within the 3′untranslated regions (3′UTRs) of target RNAs^13^. Appropriate docking of the RISC initiates transcript degradation or translation repression of the targeted mRNA^13,17^.

In vertebrates, investigations more recently aim to understand the functional complexity within families of miRNAs sequences, which may share a similar seed sequence but possess a large variety of sequence variants. In zebrafish (*Danio rerio*), members of the miR-10 family cooperate with closely positioned *Hox* gene clusters that enable a greater precision in anterior-posterior body patterning^18^. The genomic proximity of miR-10 miRNAs to their target *Hox* genes further supports the critical importance of gene silencing and post-transcriptional regulation in highly evolutionary conserved processes.

Another miRNA family, miR-430, has been shown to function in the degradation of maternally packaged transcripts to support a rapid and efficient maternal-to-zygotic transition (MZT) as the embryonic genome becomes active and assumes control over the developmental process^19^. Maternal mRNAs that are packaged into the egg prior to fertilization are known to control numerous processes during early vertebrate development, including cleavage, blastulation, and formation of the embryonic axis^20^. By definition, the activity of these transcripts must be regulated by post-transcriptional processes such as alterations in translational efficiency or mRNA stability. Members of the miR-430 family are known to target maternal mRNAs and block their expression at the MZT in order to avoid conflict between maternal and zygotic gene regulation^17,19^.

**Figure 1:**
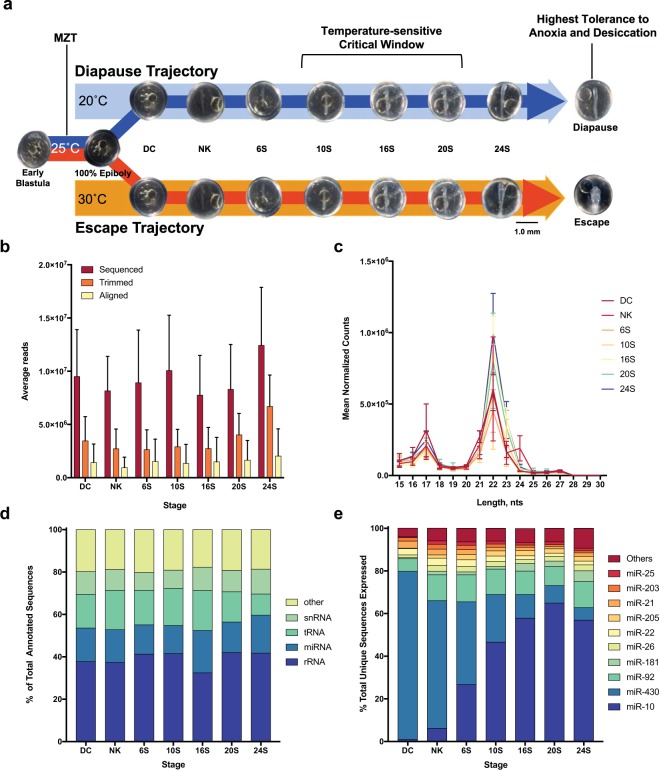
Experimental induction of alternative developmental phenotypes and resulting characteristics of the small RNA transcriptomes for embryos of *A. limnaeus*. (**a**) Groups of embryos were incubated at 25 °C until 4 dpf (100% epiboly), when they were transferred to either 20 °C or 30 °C. All stages of development sampled for this study occur after the maternal-to-zygotic transition (MZT) (which occurs just prior to epiboly) and encompass the critical window of development (approximately 10–20S) when embryos commit to a phenotypic trajectory. (**b**) Average number of reads per library for small RNA sequencing, trimming, and annotation. Bars are means ± sem (**c**) Length distribution of small RNA reads for all treatment groups. Data are plotted as means ± sem. (**d**) Categories of small RNAs annotated by miRBase and RFAM across all developmental stages. Values represent a percent of the total number of annotated small RNAs. (**e**) Contribution of the 10 most abundant annotated small RNAs as a percent of the total number of unique RNAs expressed in each developmental stage. DC, dispersed cell stage; NK, neural keel; 6S, 6 somite embryo; 10S, 10 somite embryo; 16S, 16 somite embryo; 20S, 20 somite embryo; 24S, 24 somite embryo.

The development of *A. limnaeus* and other annual killifishes is unique among vertebrates because two alternative developmental trajectories that differ morphologically, physiologically, and biochemically are known to exist^21–23^. One trajectory leads to discontinuous development and the production of embryos that enter into a profound state of metabolic depression known as diapause, while the other trajectory supports continuous development to the pre-hatching stage in embryos that “escape” arrest in diapause (Fig. 1a). Development along these two trajectories can be controlled by both maternal provisioning and embryonic incubation temperature; an incubation temperature of 30 °C induces 100% escape embryos while 20 °C induces 100% diapausing embryos^21,24^. The mechanisms by which temperature can alter gene expression and developmental trajectory are unknown.

In the current study, we explore miRNA expression along the two developmental trajectories to identify candidate regulators of known phenotypic differences. We propose that environmental signals received by the developing embryo may induce expression of specific miRNAs that target maternally packaged sequences and thus alter developmental trajectory. Importantly, this proposed regulatory mechanism allows for developmental trajectory to be regulated across multiple life stages and integrates both maternal and zygotic information. Using high-throughput RNA sequencing, we generated detailed small ncRNA transcriptomes for embryos at the same developmental stage that are developing along the two developmental trajectories as controlled by incubation temperature. This global analysis identifies a large contribution by miRNAs to the total small ncRNA transcriptome during early development. Specifically, the small ncRNA transcriptome is dominated by miR-430 and miR-10 family genes. To further test the possible contribution of miR-430 as a regulator of developmental phenotype, we performed oligonucleotide microinjections of miR-430 agonists and antagonists. This study documents the diversity of small ncRNAs during early development, and suggests a possible role for miR-430 family members in the environmental determination of developmental phenotype in embryos of *A. limnaeus*.

## Results

### Small RNA transcriptome

Embryos were reared in groups at 30 or 20 °C to induce escape and diapause trajectories (respectively) and sampled at seven morphological stages of development (Fig. 1a). After processing the small RNA sequence reads, including trimming and alignment to the genome, our methods detected approximately 350,000 to 600,000 unique sequences across all developmental stages with ≥2 normalized counts per library (Fig. 1b, Supplementary Tables S1 and S2). The length distribution of unique small RNAs identified two highly abundant size classes with peaks at 22 and 17 nts (Fig. 1c). These transcriptomes were annotated to databases of known small RNAs and further categorized as ribosomal RNA (rRNA), miRNA, transfer RNA (tRNA), or small nuclear RNA (snRNA) among many others (Fig. 1d). Across developmental stages, miRNAs represented 13–20% of annotated sequences. As a major contributing RNA class to post-transcriptional regulation of mRNA, we analyzed these sequences in further detail. The most abundantly expressed miRNA annotations (normalized counts) across all developmental stages were miR-430 and miR-10 variants (Fig. 1e). Other highly abundant miRNAs include miR-92, and miR-181.

### *A. limnaeus* miR-10 family genes

**Figure 2:**
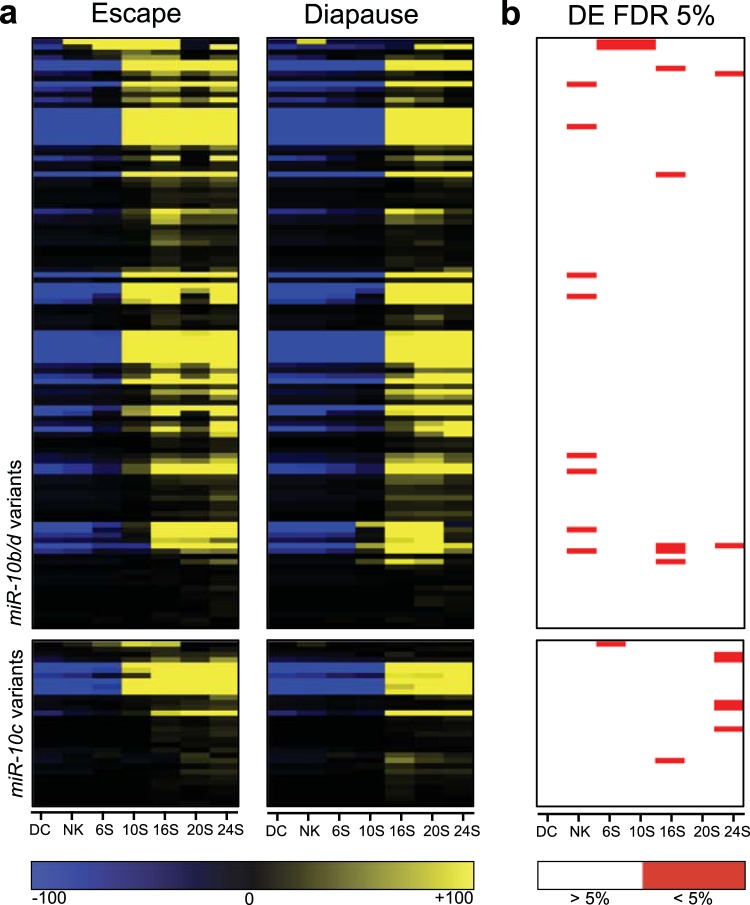
Expression of alim-miR-10 family variants. (**a**) Heat maps of median-centered normalized expression values across development for both developmental trajectories of all alim-miR-10 variants. Yellow indicates expression above the median while blue indicates expression below the median (median expression is black). Each row represents an individual variant. Upper panels are variants belonging to alim-miR10b/d and lower panels are variants of alim-miR-10c. (**b**) Heat map of statistical significance (5% FDR adjusted *p* values) of differential expression (DE) between phenotypes for each gene and stage of development. DC, dispersed cell stage; NK, neural keel; 6S, 6 somite embryo; 10S, 10 somite embryo; 16S, 16 somite embryo; 20S, 20 somite embryo; 24S, 24 somite embryo.

A total of 144 sequences across all developmental stages were annotated by miRBase as variants of alim-miR-10, and contributed to the most abundantly expressed family detected in the present study. The expression of these variants increases substantially during somitogenesis in *A. limnaeus*, contributing to over 50% of annotated reads in embryos with 16 pairs of somites (S) and greater (Fig. 1e). qPCR validation of the RNA-seq data support this expression pattern for mature miR-10b/d sequences (Supplementary Fig. S1a). To investigate if the expression of miR-10 variants is specific to phenotype, we analyzed their relative expression in escape- (30 °C) and diapause-bound (20 °C) embryos (Fig. 2a). Interestingly, most variants appear to increase in abundance earlier in escape-embryos (10S) than in diapause-trajectory embryos (16S). A handful of miR-10 family variants (mostly the high abundance variants) are differentially expressed between the two developmental trajectories (FDR adjusted *p* value < 0.05; Fig. 2b).

**Figure 3:**
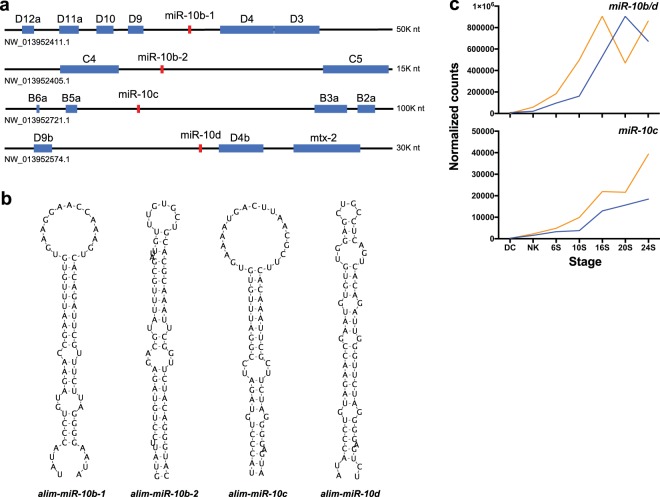
Location, structure, and total expression of alim-miR-10 family paralogs. (**a**) Genomic organization of *Hox* gene clusters and position of alim-miR-10 family members. Horizontal black lines represent individual scaffolds. Positions of alim-miR-10 genes are in red and *Hox* genes are in blue. (**b**) Predicted hairpin structures of precursor alim-miR-10 paralogs. (**c**) Transcriptomic profiles (normalized counts) of total alim-miR-10 variants separated by paralog (orange is escape trajectory; blue is diapause trajectory). DC, dispersed cell stage; NK, neural keel; 6S, 6 somite embryo; 10S, 10 somite embryo; 16S, 16 somite embryo; 20S, 20 somite embryo; 24S, 24 somite embryo.

Computational analysis of the genomic positions for alim-miR-10 family genes in *A. limnaeus* identified 4 of the 5 known variants of miR-10: b-1, b-2, c, and d (Fig. 3a). The identification and analysis of multiple alim-miR-10 family members in this study has led to a much more detailed understanding of the miR-10 family in *A. limnaeus*, and the need to reclassify a previously identified miR-10-b-3 as alim-miR-10c^24^. Comparable to what is known for other teleost species, alim-miR-10 genes are positioned within *Hox* family gene clusters (Fig. 3a). Consensus sequences of the 4 paralogs identified were highly similar and thus it is impossible to distinguish the gene origin between alim-miR-10b-1, alim-miR-10b-2, or alim-miR-10d mature sequences in our data set. The variants aligned in high abundance to 4 scaffolds of the *A. limnaeus* draft genome, NW_013952411.1, NW_013952405.1, NW_013952721.1, and NW_013952574.1, in patterns that demonstrate detection of 5′ and 3′ arms of the precursor sequences. There was no evidence of miR-10a detected in this dataset. Secondary hairpin structures were successfully predicted for miRNA precursors for these 4 paralogs further demonstrating their strong candidacy as miRNA genes (Fig. 3b). Separate profiles of expression could be characterized for the group alim-miR10-b/d and for alim-miR-10c (Fig. 3c). Both groups show an increase in abundance throughout development with overall greater expression in escape embryos. One noted exception is a decrease in alim-miR-10b/d in 20S embryos on the escape trajectory, a decrease that is not statistically significant for any of the variants (*t*-test, FDR adjusted *p* value > 0.05).

### *A. limnaeus* miR-430 family genes

**Figure 4:**
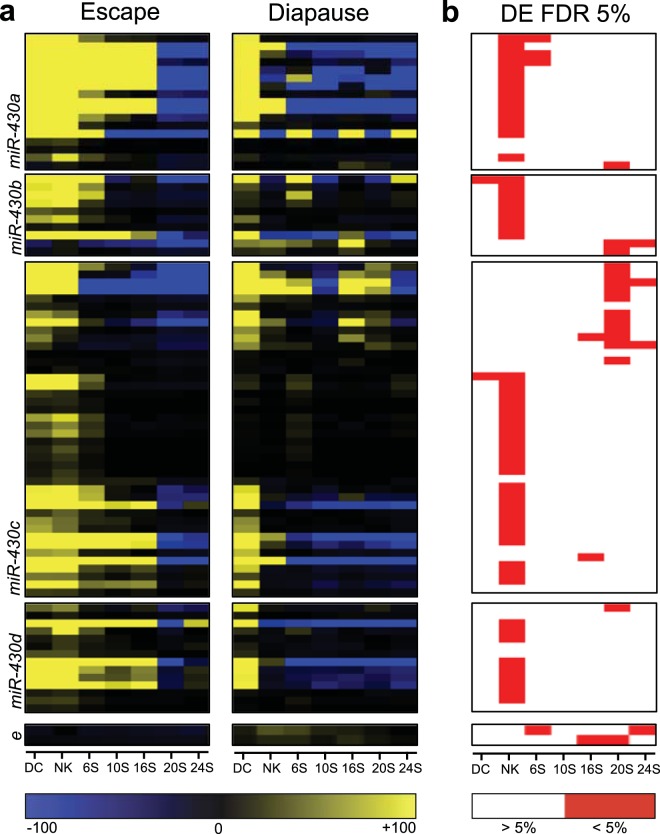
Expression of alim-miR-430 family variants. (**a**) Heat maps of median-centered normalized expression values across development for both developmental trajectories of all alim-miR-430 variants. Yellow indicates expression above the median while blue indicates expression below the median (median expression is black). Each row represents an individual variant. Panels, from top to bottom, are variants alim-miR-430-a, -b, -c, -d, and -e. (**b**) Heat map of statistical significance (5% FDR adjusted *p* values) of differential expression (DE) between phenotypes for each gene and stage of development. DC, dispersed cell stage; NK, neural keel; 6S, 6 somite embryo; 10S, 10 somite embryo; 16S, 16 somite embryo; 20S, 20 somite embryo; 24S, 24 somite embryo.

To investigate the role of the 85 sequences that annotated as alim-miR-430 family members, expression profiles for all variants were compared in embryos developing along the escape and diapause trajectories (Fig. 4a). A majority of alim-miR-430 mature sequences were highly abundant during early development and declined substantially during somitogenesis. qPCR validation of the RNA-seq data support this expression pattern for mature miR-430a sequences (Supplementary Fig. S1b). MiR-430 sequences have a significantly higher abundance in escape trajectory embryos especially at the neural keel stage of development (FDR adjusted *p* value < 0.05; Fig. 4b).

**Figure 5:**
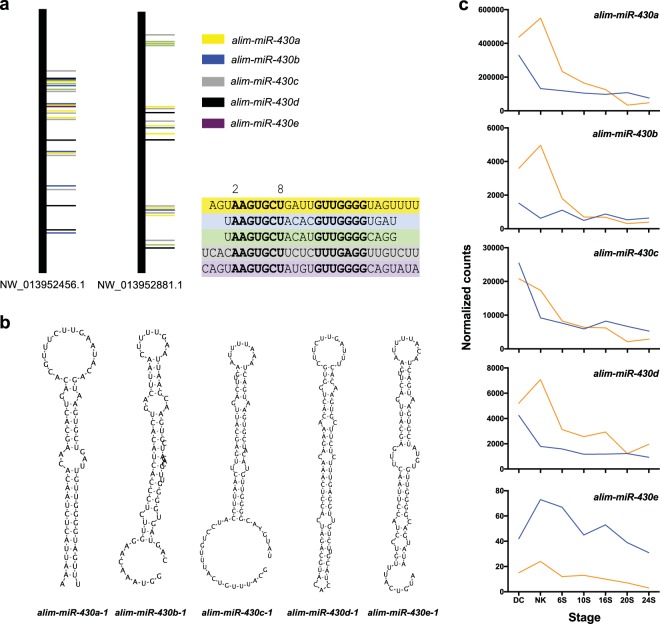
Location, structure, and total expression of alim-miR-430 family paralogs. (**a**) Genomic organization of the two large clusters of alim-miR-430 genes and the position of family member paralogs. Vertical black lines represent individual scaffolds. The positions of alim-miR-430 genes are color coded by paralog and represented by horizontal bars. (**b**) Predicted hairpin structures of precursor alim-miR-430 paralogs. (**c**) Transcriptomic profiles (normalized counts) of total alim-miR-430 variants separated by paralog (orange is escape trajectory; blue is diapause trajectory). DC, dispersed cell stage; NK, neural keel; 6S, 6 somite embryo; 10S, 10 somite embryo; 16S, 16 somite embryo; 20S, 20 somite embryo; 24S, 24 somite embryo.

Analysis of the genomic positions for alim-miR-430 family genes identified 5 possible variants that mapped to two large scaffolds, NW_013952456.1 and NW_013952881.1, in the *A. limnaeus* draft genome assembly (Fig. 5a). All variants were investigated for the ability to form hairpin secondary structures (Fig. 5b) and named as paralogs a-e of the alim-miR-430 gene family. The consensus sequences of the 5 paralogs share a 5′- and 3′- end sequence but exhibit a unique 4-mer in the central part of the sequence (Fig. 5a). Each paralog can be further identified by isoforms within the paralog family. Mature sequences of the 5 alim-miR-430 paralogs of *A. limnaeus* were evaluated for variant-specific expression profiles (Fig. 5c). The sequences belonging to alim-miR-430a are highly abundant in comparison to other family members by 1–2 orders of magnitude in most cases. This variant, along with alim-miR-430b and alim-miR-430d had a greater abundance at the dispersed cell (DC), neural keel (NK), and 6S stages in embryos developing on the escape trajectory in comparison to the diapause trajectory. In contrast, expression of alim-miR-430c was similar in the two trajectories, while variants of alim-miR-430e demonstrated a strong diapause-specific expression pattern. All paralogs decline in abundance during early development and all but the miR-430e family members reach roughly equivalent abundance in 20S embryos or later.

**Figure 6:**
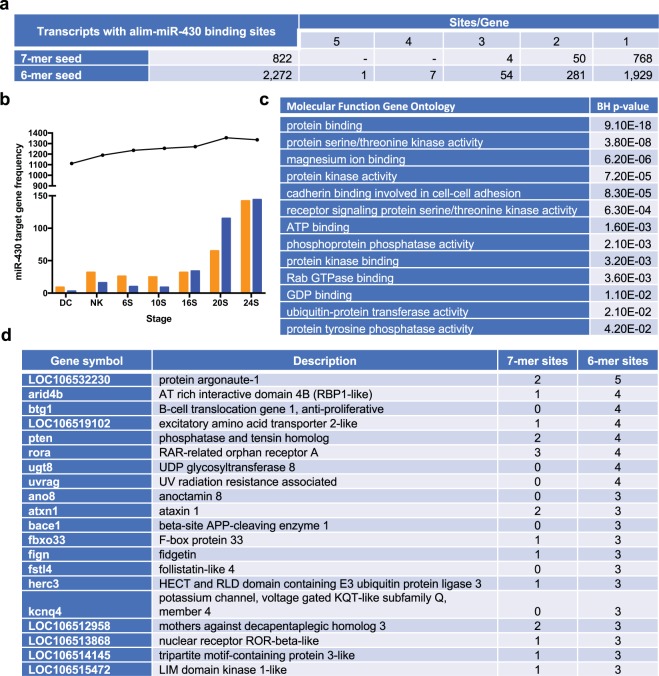
Summary of potential alim-miR-430 gene targets. (**a**) Number of *A. limnaeus* gene transcripts with potential binding sites for miR-430 seeds (6-mer and 7-mer) within the 3′UTR. (**b**) The number of potential alim-miR-430 targets increases during development. The black line represents the number of expressed mRNA sequences with predicted alim-miR-430 binding sites in each developmental stage. The colored bars represent genes with alim-miR-430 binding sites that are differentially expressed in the two developmental phenotypes (orange is escape, blue is diapause trajectory). DC, dispersed cell stage; NK, neural keel; 6S, 6 somite embryo; 10S, 10 somite embryo; 16S, 16 somite embryo; 20S, 20 somite embryo; 24S, 24 somite embryo. (**c**) Functional annotation of potential targets of miR-430 with molecular functions statistically enriched and having Benjamini-Hochberg adjusted *p* values < 0.05. (**d**) Genes in *A. limnaeus* genome with the most binding sites. mRNA transcript data are from^56^.

Potential gene targets were identified in the *A. limnaeus* genome with complementary binding sites within the 3′UTR for the 6- and 7-mer miR-430 seed sequences (Fig. 6a). The number of potential target genes increases during development (Fig. 6b). These potential target genes were enriched with molecular functions of protein interactions including binding, signaling, and regulatory roles (Benjamini-Hochberg adjusted *p* values < 0.05; Fig. 6c). The mean lengths of 3′UTRs across developmental stages do not appear to contribute to the presence of miR-430 binding sites (Supplementary Fig. S2). Gene transcripts with an increase in the number of instances of binding sites do not differ significantly in 3′UTR length (ANOVA; *p* > 0.05) and mean lengths of 3′UTRs with any binding sites do not differ across development (ANOVA; *p* > 0.05). Gene targets for miR-430 often display multiple binding sites^17^, and thus we used the 6-mer binding site data to be as inclusive as possible. Interestingly, the transcript with the most binding sites comes from the gene *argonaute* which encodes for a protein critical in small RNA silencing mechanisms (Fig. 6d). Other genes of interest with a high number of binding sites include the UV radiation resistance associated gene (*uvrag*) and nuclear receptors such as retinoic-acid-receptor (RAR) related orphan receptor A (*rora*) and nuclear receptor ROR-beta-like, (LOC106513868). The potential gene targets for alim-miR-430 variants represent putative key regulators of normal development, and trajectory-specific regulation of development that will require further functional analysis (see Supplementary Table S3 for the full list).

#### Microinjection of oligonucleotides

The RNA microinjection protocol resulted in high survival rates (80–90%) in 1–2 cell stage embryos based on multiple rounds of microinjections with an alim-miR-430a mimic, an alim-miR-430a knockdown morpholino oligonucleotide, and a scramble morpholino oligonucleotide. Delivery and persistence of the RNA was confirmed via fluorescence microscopy.

**Figure 7:**
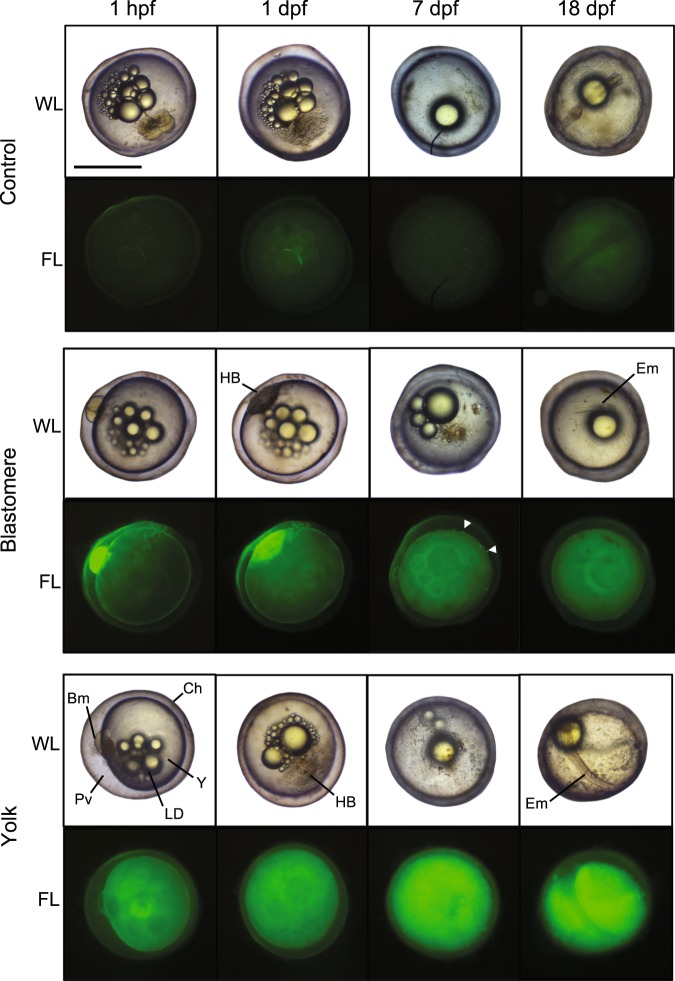
Phenotypes of embryos injected with alim-miR-430a mimic morpholino oligonucleotides. Embryos were injected with 500 nM dsRNA at 1 hour post fertilization (1 hpf) into either the yolk directly under the forming blastomere (yolk), directly into the first blastomere (blastomere), or were not injected (control). Embryos were observed under white light (WL) and fluorescent light (FL) throughout development at 1, 7 and 18 dpf. Control embryos were at the high blastula stage at 1 dpf, in reaggregation at 7 dpf, and in mid-somitogenesis at 18 dpf. Injected embryos were delayed in their development. White arrow heads indicate dispersed cells with positive fluorescent staining. Scale bar = 1 mm. Ch, chorion; Bm, blastomere; HB, high blastula; Em, embryo; LD, lipid droplets; Pv, perivitelline space; Y, Yolk.

Injections of oligonucleotides into the yolk did not lead to fluorescent labeling of the embryonic cells, but the yolk maintained a strong and stable signal for up to 18 days post-fertilization (dpf) and longer after injection at fertilization (Fig. 7). Injection into the blastomeres lead to a strong fluorescence signal within the embryonic cells that persisted until epiboly, after which the fluorescent signal was lost (Fig. 7). Injection of both the alim-miR-430a mimic and knockdown oligonucleotide failed to alter developmental trajectory in a consistent manner (data not shown).

## Discussion

This study is the first to characterize the small ncRNA transcriptome associated with development along the two developmental trajectories possible in embryos of annual killifishes. Consistent with previous work in this species, we have identified miRNAs as major contributors to the small ncRNA transcriptome during development with the most abundant sequences annotating to conserved families of vertebrate miRNAs including miR-10, miR-430, miR-92, and miR-181^24,25^. Many families of miRNAs have been implicated in the regulation of gene expression in a variety of contexts during embryonic development^8,12,13,26–28^. Two of which, miR-10 and miR-430 are highly abundant and differentially expressed during early development in *A. limnaeus*. In addition to miRNAs, we found a large abundance of RNAs at the length of 17 nts that we were unable to annotate from known small ncRNA databases. This particular class size of RNA is relatively underrepresented in the literature and only shows resemblance to what can be described as small RNAs derived from small nucleolar RNAs (snoRNAs) that range from 17 to 19 nts in length^29^. Their role may likely contribute to RNA processing mechanisms similar to other ncRNA however any patterns of induction in response to temperature have yet to be explored.

The miR-10 family has been extensively investigated and characterized by its co-evolution with, and genomic proximity to, *Hox* family clusters^30,31^. *Hox* genes code for highly conserved transcription factors that are crucial for anterior-posterior patterning during vertebrate development. Additionally, mounting evidence has demonstrated both the co-expression of miR-10 and *Hox* genes during development and the targeting of *Hox* transcripts by miR-10 family members^18,32^. In *A. limnaeus* embryos there is a sharp increase in miR-10 variant expression at the 10S stage in escape embryos incubated at 30 °C. A similar sharp increase is observed in the diapause-bound (20 °C) embryos at the 16S stage. These expression patterns are consistent with a role for miR-10 family members in helping to establish the anterior and posterior axis and develop appropriate structures during this time. The earlier expression of miR-10 variants in the escape trajectory embryos suggests the possibility of heterokairy in gene expression in the two developmental trajectories and warrants further investigation.

Only a few of the miR-10 variants were differentially expressed in the two developmental trajectories. Of note are variants that are highly abundant in escape embryos starting at the NK stage through the 16S stage. These same variants are expressed at much lower levels in diapause-bound embryos during this window of development. Another interesting feature of miR-10 variants is their known ability to *increase* the translation of proteins involved in the support of protein synthesis, especially ribosomal proteins^33^. This role is rather unique among miRNAs which typically block translation of proteins. Protein synthesis is known to be depressed in embryos developing along the diapause trajectory^34^, and perhaps the reduced expression of these miR-10 variants could lead to reduced translation of ribosomal proteins and help to explain the limited ability of these embryos to support high levels of protein synthesis. Alternatively, differential expression could be a mechanism for buffering the potential perturbing effects of temperature on developmental timing of *Hox* gene expression in order to support normal development.

Members of the miR-430 family are known to be expressed at the MZT which occurs in most vertebrates prior to axis formation. In *A. limnaeus* the MZT occurs around the initiation of epiboly during a time known as the midblastula transition^35^. Interestingly, miR-430 expression is still high in dispersed cell stage embryos several days after the midblastula transition has occurred. Further, many miR-430 family variants are highly expressed during early somitogenesis in the escape-bound embryos. This prolonged high abundance and differential expression between the two developmental trajectories is unique compared to other models of fish development. Interestingly, the number of potential miR-430 targets are higher in escape embryos early in development until the 20S stage, when they become more abundant in the diapause-bound embryos. This shift in available targets suggests a clearing of miR-430 targets from the escape-bound embryos. However, it is also possible that miR-430 may be contributing to gene expression in both phenotypes and targeting different transcripts in either scenario.

While predicting targets of miRNAs by sequence homology can be imprecise and is known to be somewhat unreliable, it is still the most common way to interrogate the possible effects of a miRNA at the genomic level. The fact that miRNAs are highly conserved in sequence and function across vertebrate taxa allows for the generation of hypotheses for functions of miR-430 in *A. limnaeus*. Gene targets of miR-430 in other species have been predicted, and many potential targets are critical regulators of development including members of the Wnt and TGF-β/Nodal signaling pathways^19,36,37^. Interestingly, the TGF-β pathway is a known regulator of dormancy in *C. elegans* dauer larvae^38^, and a maternally-packaged TGF-β receptor contains a miR-430 binding site in *A. limnaeus*. However, there are many genes in *A. limnaeus* (2,272) that have predicted binding sites for miR-430. An interesting population of transcription factors occupies the list of genes with the most binding sites. In addition, other genes that are important to developmental timing were discovered as potential targets including a member of the POU family of transcription factors (LOC106534879), period circadian clock 1 (*per1*), multiple members of forkhead box genes, and multiple members of cell cycle regulating cyclin genes (Supplementary Table S3).

Because developmental trajectory can be influenced by both maternal cues and embryonic incubation temperature^21^, we investigated if temperature-induced differences in miR-430 variant abundance could target maternal transcripts that may be critical for programming embryos to develop along the diapause trajectory. A number of trajectory-specific mRNA splice variants were identified to be maternally packaged^24^. Two of these variants contain predicted miR-430 targets sites: sideroflexin-5 like (LOC106524277) and phosphorylase kinase, beta (*phkb*). Sideroflexin-5 belongs to a family of mitochondrial tricarboxylate carrier proteins and is known to contribute to brain development in *Xenopus laevis*^39^. This is the first report to our knowledge of any member of the sideroflexin family being a potential miR-430 target. The exact function of sideroflexin-5 is unknown, but it has been shown to transport citrate under *in vitro* conditions. Diapause-bound embryos are known to be poised for anaerobic metabolism based on high ratios of lactate dehydrogenase activity compared with citrate synthase activity^40^. A role for a diapause-specific citrate transporter is an intriguing possibility that deserves future attention. Another splice variant that may be targeted by miR-430, *phkb*, is highly upregulated in diapause embryos at fertilization^24^. This particular gene is a known contributor to regulation of glucose mobilization from glycogen that can be regulated by insulin and insulin-like signaling pathways^41^. Reduced insulin-like signaling has been established as an important contributor to the diapause trajectory in *A. limnaeus*^23^, and perhaps a diapause-specific variant of *phkb* is required to support the unique metabolic poise of diapausing embryos that would be incompatible with the high metabolic demands of the escape trajectory. In light of these findings, we propose that key maternally packaged mRNA transcripts inherited by diapause-bound embryos could be targeted by zygotic miR-430 in response to environmental cues to override maternal signals.

The systemic impact of miRNAs is commonly investigated by either the removal of the miRNA processing enzyme, *dicer*, or by the targeted silencing of mature miRNAs^42,43^. It has been shown that mutant embryos lacking functional *dicer* display morphological defects during gastrulation and brain development in zebrafish. The reintroduction of miR-430 rescues these defects, suggesting that a loss of miR-430 alone is responsible for these phenotypic outcomes^42^. Therefore, we approached our functional analysis of miR-430 by attempting to enhance or decrease the expression of one of the most abundant miR-430 variants, alim-miR-430a, in embryos of *A. limnaeus*. These microinjection experiments did not alter developmental trajectory as predicted. However, at this point it is unclear if this negative result is due to a lack of a role for miR-430a in determining developmental trajectory, or the unanticipated ability of the embryos to purge the foreign RNA from the embryonic cells prior to the temperature-sensitive critical window of development when we would expect miR-430 to act. This suggests the embryos have a mechanism for either purging or degrading foreign RNA from cells, or perhaps for removing cells containing foreign RNA. Dispersion and reaggregation of the embryonic blastomeres is a developmental process unique to annual killifishes where the deep blastomeres lose their attachments to other cells and migrate across the yolk. Previous studies suggest that this stage of development can buffer against UV-C-induced DNA damage in order to support normal development even in stressful and damaging conditions^44,45^. It is possible that high levels of foreign RNA may induce similar buffering mechanisms in these embryos and prevent the full action of the oligonucleotides. Based on the results of the knockdown and mimic experiments, future experiments will require transgenic or transient expression methodologies to ensure proper RNA dosage and persistence in *A. limnaeus* embryos^46,47^.

In summary, this study suggests a role for temperature-induced miRNA regulation of developmental phenotype in embryos of *A. limnaeus*. While many additional studies are needed to fully support this hypothesis, the expression patterns and potential targets of miR-430 family members described in this study suggest a role for this miRNA in the integration of environmental signals to alter developmental trajectory. The embryonic alteration or regulation of maternally provisioned genes could provide this species with a strategy to override inherited phenotypic cues in an environment that often fluctuates between tolerable and intolerable conditions. These data suggest a tangible molecular mechanism for maternal-zygotic conflict in life history decisions that could have far-reaching implications beyond the annual killifish model. Further investigations of the role of miRNAs in regulating alternative trajectories may shed light on the role of maternal packaging in the regulation of phenotype, and lead to a better understanding of the role of epigenetic gene regulation in vertebrate development.

## Methods

### Experimental design

Adult fish were cared for and embryos were collected as previously described^48^. Embryos were collected and pooled together from 42 pairs of fish and maintained for 4 dpf in an incubator at 25 °C in darkness^48^. Once reaching 100% epiboly, embryos were transferred to incubation temperatures of 20 °C to induce the diapause phenotype and 30 °C to induce the escape phenotype^21^. Embryos were maintained at their experimental temperature until being flash-frozen at seven morphological stages of development that cover the relevant window when the commitment to either the escape or diapause trajectory is determined: DC stage (24 h after temperature transfer), NK, and 6S, 10S, 16S, 20S, and 24S^35^ (Fig. 1a). Groups of embryos (20–40 embryos per sample) were used for RNA extraction and small RNA-sequencing (n = 6 samples per stage, n = 3 samples per treatment).

### Small ncRNA transcriptomes

The details of RNA extraction, small RNA sequencing, and bioinformatics analysis of small ncRNA data have been described previously in Romney and Podrabsky^24^. The details of the samples used and the bioinformatics results of the small ncRNA profiles are available in Supplementary Table S1.

### Quantitative real-time PCR (qPCR)

Relative expression of mature miR-430a and miR-10b/d variants were quantified in a subset of the 30 °C samples using miScript qPCR technology according to the manufacturer’s instructions (Qiagen, Redwood City, CA). First strand synthesis was conducted using 1 μg of total RNA as starting material in a total reaction volume of 20 μl using the HiFlex buffer. Reactions were incubated for 60 min at 37 °C followed by 5 min at 95 °C to inactivate the reverse transcriptase enzyme. Single-stranded cDNA was diluted 1:10 with reagent grade water and then used as template for the miScript SYBR green qPCR kit (Qiagen). Custom primer assays were designed by Qiagen for the miR-430a sequence (AGUAAGUGCUGAUUGUUGGGG) and miR-10b/d sequence (UACCCUGUAGAACCGAAUUUGCG). Reactions were run on an Agilent Mx3005P qPCR system for 40 cycles according to the instructions provided in the Qiagen kit. All reactions were tested for a single product using melting curve analysis. Threshold values (C_t_) were calculated by Agilent MxPro qPCR software. Expression data were normalized to total RNA and are reported as expression relative to embryos in the DC stage.

### Analysis of alim-miR-430 and alim-miR-10 family genes

Abundance patterns of alim-miR-430 and alim-miR-10 family sequences were organized and visualized using hierarchical clustering and heat maps of median-centered (by sequence) abundance data expressed as normalized counts. Clustering was accomplished using uncentered Pearson correlation with average linkage in Gene Cluster 3.0^49,50^. Heat maps were generated with Java Treeview 1.1.6r4^51^. Mature sequences that annotated as either alim-miR-430 or alim-miR-10 variants by miRBase were used to investigate the organization of the genes for these transcripts in the *A. limnaeus* genome. Positions for miRNA genes were identified by the alignment of mature sequences with perfect match to the draft assembly of the NCBI *A. limnaeus* genome (1.0; GenBank accession GCA_001266775.1) with reference to the genome annotation Release 100^52^. The majority of individual variants, with only a few exceptions, aligned in clusters of similar miRBase annotations (paralogs, for example: alim-miR-430b, alim-miR-430c), which aided identifying *A. limnaeus* specific paralogs. Stretches of expanded genomic regions representing putative miRNA genes were evaluated for hairpin secondary structures using the Vienna package RNAfold prediction tool from Geneious (R 8.1.6). These potential miRNA genes were annotated as *A. limnaeus*-specific paralogs from the alim-miR-430 or alim-miR-10 family and were used for identifying variant-specific expression profiles.

### Identification of putative gene targets for miR-430 sequences

Seed sequences of 6 and 7 nts in length (6- and 7-mer, respectively) on the 5′ arm of the alim-miR-430 family members were determined as: AAGUGCU and AGUGCU. The complement of these sequences was identified within the 3′ UTR of all coding genes in the *A. limnaeus* genome by inferring the differences between exon and gene CDS features annotated by NCBI using a python script available at (ftp://ftp.ncbi.nlm.nih.gov/genomes/TOOLS/add_utrs_to_gff/). Functional annotation of predicted miR-430 gene targets was determined by generating protein homologs for all gene identities to *H. sapiens* gene IDs using BLASTp with a threshold e value less than 1.0E-5. Using the Database for Annotation, Visualization and Integrated Discovery (DAVID) bioinformatics software, clusters of *A. limnaeus* genes that were identified by a *H. sapiens* homolog gene ID, were tested for GO term enrichment with Bejamani-Hochberg adjusted *P* values < 0.05.

### Microinjection of oligonucleotides

A microinjection protocol for the effective introduction of RNA molecules into *A. limnaeus* embryos was developed by adaptation of methods described for other fish species^46,53–55^. Fertilized eggs were embedded into a petri dish of 1% w/v low melting point agarose at 28 °C and cooled to its gelling point at 24 °C. Embryos were visualized using a dissecting microscope at 20–40X total magnification. Microinjections were performed using an MPPI-3 Pressure injector (Applied Scientific Instrumentation, Inc., Eugene Oregon) attached to a MX130 4-axis micromanipulator (Siskiyou Corporation, Grants Pass, OR). Embryos at the 1–2 cell stage were either injected with 1–2 pulses into the yolk mass or directly into the blastomeres with an alim-miR-430a mimic (see below). Afterwards, embryos were briefly stored in the dark for 1–3 hours before being removed from the agarose and transferred to incubation media and maintained at 25 or 30 °C for further observations depending on the experiment.

Microinjection needles were prepared using borosilicate glass capillaries with an internal filament (1.0 mm O.D. and 0.58 mm I.D.). Capillaries were pulled into micropipette needles using a Sutter Micropipette Puller, Model P-80/PC (California, USA). Similar to previous reports for other killifish species, the needle profiles that are best for penetrating *A. limnaeus* chorions have a shorter and more robust tip than that of a typical zebrafish microinjection needles^46^. The program used has two loops with the following parameters: Heat: 700, Pull: 50, Velocity: 20, Time: 44.

A morpholino oligonucleotide sequence for alim-miR-430a expression inhibition was designed and manufactured by Gene Tools (Philomath, OR) in the reverse complement for alim-miR-430a: 5′-ACTACCCCAACAATCAGCACTTACT-3′, and labeled with a 3′-carboxyfluorescein. The standard fluorescein-labeled control oligonucleotide sequence offered by Gene Tools was used as an injection control: 5′CCTCTTACCTCAGTTACAATTTATA-3′. An RNA mimic to induce enhanced alim-miR430a activity was manufactured by Integrated DNA Technologies as an RNA duplex oligonucleotide with one arm identical to the mature seq of alim-miR-430a: 5′-AGUAAGUGCUGAUUGUUGGGGUAG-3′ (active anti-sense strand) and the arm as 3′-TCAUUCACGACUAACAACCCCA-5′ labeled with a 5′ 6-FAM™ fluorophore. All oligonucleotides were diluted to either 500 nM or 750 nM injection stocks in RNase-free water.

### Ethics approval and consent to participate

Adult *A. limnaeus* were cared for and embryos were collected and incubated according to standard laboratory methods established for this species^48^ under approval of the PSU IACUC (Protocol #33).

## Electronic supplementary material

Supplementary Information

Dataset 1

Dataset 2

Dataset 3

## Data Availability

The original data sets are available via NCBI’s Sequence Read Archive (SRA): https://www.ncbi.nlm.nih.gov/bioproject/PRJNA272154. The accession identity of each NCBI biosample used in this study can be found in Supplementary Table S1.
