# Supplementary material for: Small noncoding RNA profiles along alternative developmental trajectories in an annual killifish
Source: Sci Rep. 2018 Sep 6;8:13364. doi: 10.1038/s41598-018-31466-7 (PMC6127099; doi:10.1038/s41598-018-31466-7)
Supplement: Supplementary file 1 — Supplementary Information [file 41598_2018_31466_MOESM1_ESM.pdf]

Supplementary Information for:

Small noncoding RNA profiles along alternative developmental trajectories in an annual killifish

Amie L. T. Romney[1,2], Jason E. Podrabsky[1]

[1]Department of Biology, Portland State University, P.O. Box 751, Portland OR 97207

[2]Department of Anatomy, Physiology & Cell Biology, University of California at Davis School of Veterinary Medicine, One Shields Ave, Davis, CA 95616

Amie L. T. Romney: arom2@pdx.edu

Jason E. Podrabsky: jpod@pdx.edu

Included:

Supplementary Figure S1. qPCR validation of RNAseq data

Supplementary Figure S2. Distributions of 3' UTR lengths with miR-430 binding sites.

Also available online as separate files:

Supplementary Table S1. Tissue sampling, RNA extraction, and bioinformatic details of small RNA transcriptomic profiling in embryos of *A. limnaeus*.

Supplementary Table S2. Normalized counts of small noncoding RNA molecules identified in embryos of *A. limnaeus*.

Supplementary Table S3. Potential mRNA gene targets with miR-430 binding sites in the *A. limnaeus* genome.

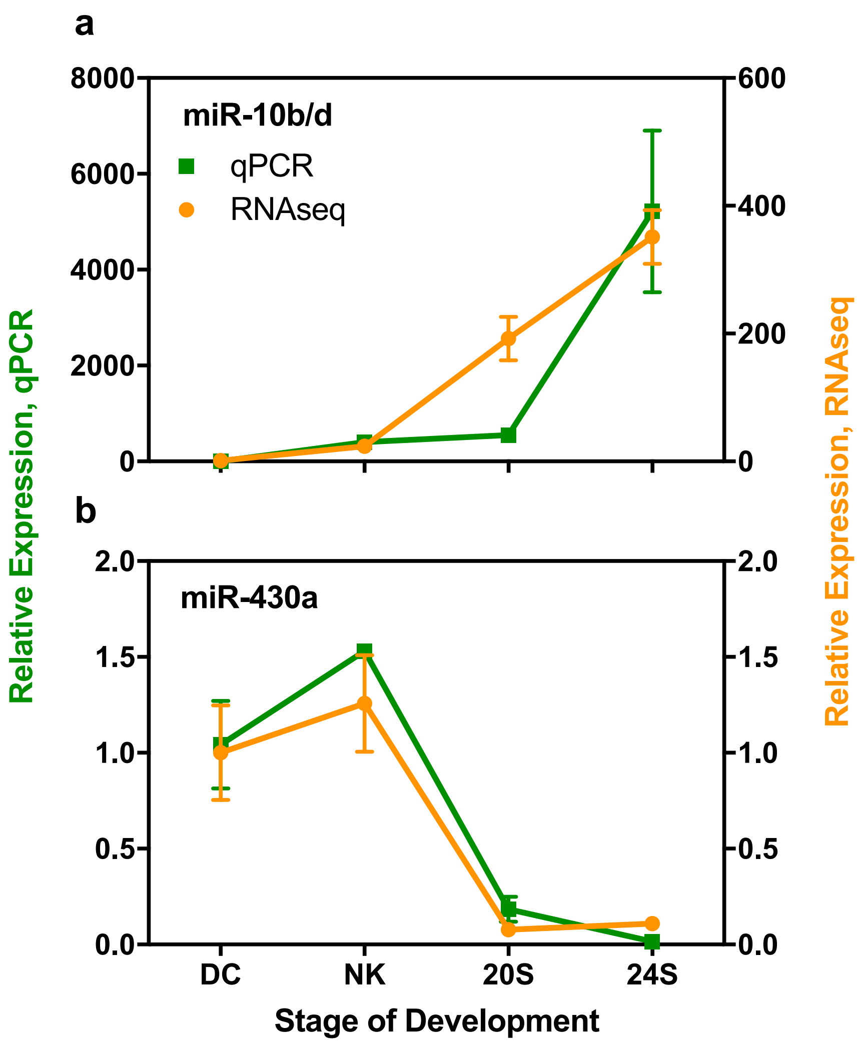

Figure S1. qPCR validation of RNAseq data. Patterns of gene expression are similar when compared using qPCR and RNAseq techniques for mature miRNA sequences of both (a) miR-10b/d and (b) miR-430a. Data are for embryos developing along the escape trajectory at 30°C. Orange represents RNAseq data presented in the main body of the manuscript, while green represents qPCR data for the same RNA samples. All data are expressed relative to the mean expression levels in the earliest developmental stage, dispersed cell embryos. DC = dispersed cell stage; NK = neural keel; 20S = 20 somite pairs; 24S = 24 somite pairs. Data are means ± sem.

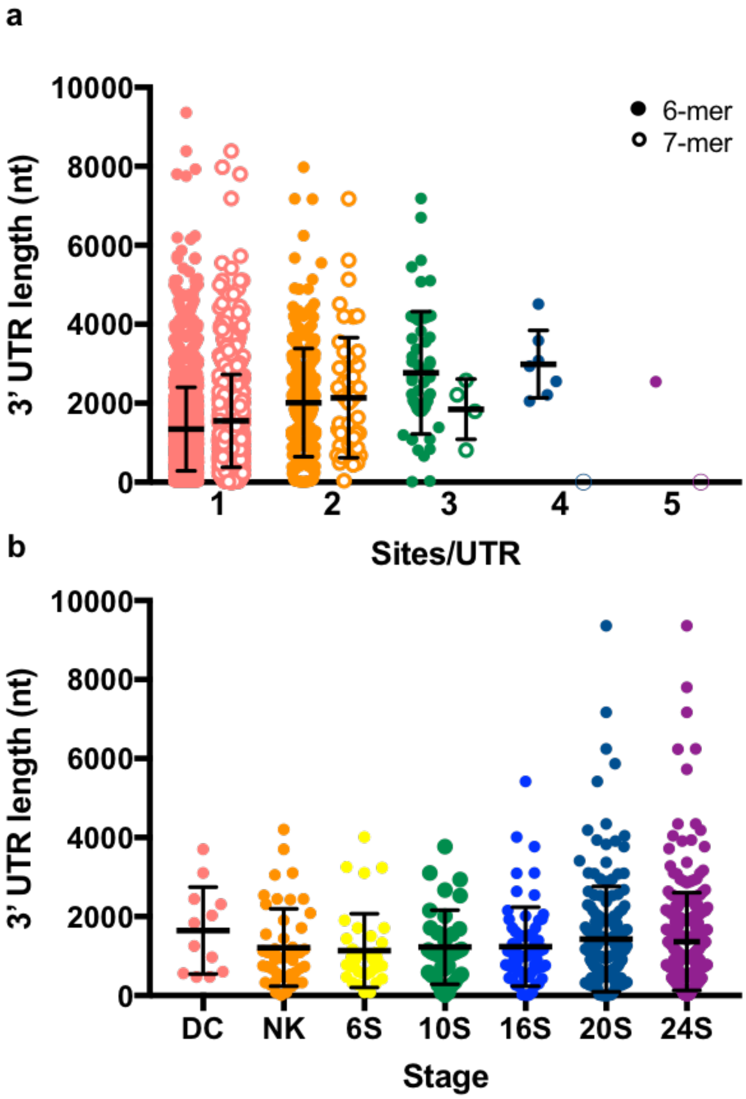

Figure S2. Distributions of 3' UTR lengths with miR-430 binding sites. (a) Mean lengths of gene transcript 3' UTRs (nt) with instances of hexamer (6-mer; solid circles) and heptamer (7-mer; hollow circles) miR-430 binding sites. (b) Mean lengths of 3' UTRs containing at least one miR-430 6-mer binding site for those gene transcripts that are significantly enriched in either the escape or diapause phenotype at each developmental stage. Lines represent means ± S.D. for each group. There are no statistically significant trends in 3'UTR length for sites/UTR, or by developmental stage (ANOVA, *p* value > 0.05)
